# Comparative analysis between abdominal aortic aneurysm and popliteal artery aneurysm

**DOI:** 10.1016/j.jvssci.2024.100279

**Published:** 2024-12-28

**Authors:** Marcos Vinícius Melo de Oliveira, Alexandre Malta Brandão, Gina Camillo Rocha Silvestre, Alexandre Queiroz Silva, Michele Alberto Marques, Marcia Martins Reis, Maria de Lourdes Higuchi, Erasmo Simão da Silva

**Affiliations:** a Vascular and Endovascular Division, Department of Surgery, University of São Paulo Medical School, São Paulo, Brazil; b Laboratory of Cardiac Pathology, Heart Institute, University of São Paulo Medical School, São Paulo, Brazil

**Keywords:** Aorta, Abdominal, Popliteal artery, Aneurysm, Biomechanics, Immunohistochemistry

## Abstract

**Objective:**

Infrarenal abdominal aortic aneurysm (AAA) and popliteal artery aneurysm (PAA) are localized arterial dilatations with distinct clinical outcomes. This study aimed to comprehensively compare these two types of aneurysms' biomechanical, histological, and immunohistochemical characteristics.

**Methods:**

This study included 180 patients with AAA and 18 with PAA. Medical history and imaging data were collected. Biomechanical testing assessed arterial wall mechanical strength and elasticity, and histological and immunohistochemical analyses examined tissue composition and inflammatory markers.

**Results:**

PAA wall fragments demonstrate higher failure strain energy (13.36 N/m^2^ vs 9.95 N/m^2^; *P* = .023), a measure of mechanical strength. Regarding immunohistochemical markers, AAA exhibited more B lymphocyte cells in the adventitia (CD20 1475.50 vs 320; *P* = .003) compared with PAA. Additionally, AAA demonstrated more adipogenic differentiation in the adventitia (PPARgamma 4854.50 vs 778; *P* = .009), whereas PAA showed more adipogenic differentiation in the intima (KLF5 283.50 vs 77.50; *P* = .039).

**Conclusions:**

PAA wall fragments demonstrate greater mechanical strength compared with AAA wall fragments. In contrast, AAA walls contain a greater number of B lymphocytes within the adventitia compared with PAA walls. Adipogenic differentiation is more pronounced in the adventitia of AAA than in PAA, whereas in PAA, it is more prominent in the intima compared with AAA.

**Clinical Relevance:**

The clinical significance of this study lies in its potential to enhance our understanding of the distinct pathophysiological mechanisms underlying abdominal aortic aneurysms, which is often associated with rupture, and popliteal artery aneurysms, which are more prone to thrombosis and distal embolization. By comprehensively comparing the biomechanical, histological, and immunohistochemical aspects of these two aneurysm types, the study aims to illuminate the factors contributing to their differing clinical presentations and outcomes.

**Article Highlights**

• **Type of Research:** Single-center observational cross-sectional study
• **Key Findings:** Popliteal artery aneurysm (PAA) wall fragments demonstrate higher mechanical strength. Abdominal aortic aneurysm (AAA) wall fragments exhibit a greater number of B lymphocyte cells in the adventitia. Adipogenic differentiation is prominent in the intima of PAA and the adventitia of AAA.
• **Take Home Message:** PAA walls exhibit greater mechanical strength than AAA walls, with adipogenic differentiation predominantly localized to the intima. In contrast, abdominal aortic aneurysms show a higher presence of B lymphocytes and adipogenic differentiation in the adventitia.

Despite experiencing comparable blood pressures, the aorta and popliteal artery, distinct in structure and function, exhibit contrasting aneurysm manifestations.^1^, ^2^, ^3^ The elastic aorta, responsible to withstand pulsatile pressure, is prone to rupture in the case of an infrarenal abdominal aortic aneurysm (AAA).^4^, ^5^, ^6^, ^7^, ^8^, ^9^ In contrast, the muscular popliteal artery, responsible for blood flow regulation, is more susceptible to embolization or thrombosis when affected by a popliteal artery aneurysm (PAA).^10^ This clinical divergence underscores the importance of recognizing the inherent differences between these two arterial types in aneurysm management.^11^, ^12^, ^13^

Comparative analyses of AAAs and PAAs highlight distinct pathophysiological characteristics, despite their shared feature of tunica media degradation. Inflammatory cell infiltration is localized predominantly to the intima in PAA and the adventitia in AAA. PAA exhibits higher iron deposits and matrix metalloproteinase 2 (MMP2) activity, whereas AAA displays elevated cytokine levels.^14^ Notably, adipose tissue accumulation within the adventitia, coupled with increased adipogenic differentiation, is a unique feature of advanced AAA, potentially contributing to their rupture risk.^15^ No articles compare the biomechanical aspects of AAA and PAA.

This study aimed to comprehensively compare the biomechanical, histological, and immunohistochemical aspects of AAA and PAA.

## Methods

### Study design

This observational, cross-sectional study, conducted from January 2004 to May 2021, recruited two patient groups: those with AAA measuring ≥35 mm in diameter and those with PAA measuring ≥20 mm in diameter. Tissue samples were taken from the anterior wall of the AAA and any accessible part of the PAA during surgery at a tertiary referral hospital. All AAA <50 mm were collected during emergency surgery for ruptured aneurysms.

### Eligibility

Inclusion criteria were individuals with AAA or PAA of nonspecific cause undergoing open surgical repair, subject to consent from both the patient (or guardian) and the surgeon for arterial specimen removal. An age range of 60 to 85 years was applied owing to the known influence of age on the studied variables.^16^ Exclusion criteria were patients with aneurysms of other etiologies (eg, inflammatory or mycotic), aneurysms other than infrarenal AAA or PAA, and infectious disease.

Ruptured aneurysm status did not disqualify patients from participating in this study. During elective and urgent surgical repairs for AAA and PAA, the surgeon had the discretion to decide whether or not to collect an arterial fragment. When collecting the fragment that might delay or interfere with the surgery, the researchers explicitly instructed the surgical team not to do so. The foremost priority of the study was to ensure that it did not hinder the surgical procedure or increase the risk of complications for the patients.

### Ethics approval

All procedures performed in this study involving human participants were under the institution's ethical standards, the national research committee, the 1964 Helsinki Declaration and its later amendments or comparable ethical standards. This project was registered at Plataforma Brasil and was approved by the Institutional Review Board of the Hospital das Clinicas, University of São Paulo Medical School (HCFMUSP), and the Brazilian National Research Ethics Commission (Comissão Nacional de Ética em Pesquisa, CONEP) with protocol number 40313220.9.0000.0068.

### Clinical variables

Medical history was collected regarding smoking (current or previous), systemic arterial hypertension, and diabetes mellitus. All available ultrasound examinations and computed tomography scans from the hospital database or patient records were analyzed to determine the transverse diameter of the vessel and the precise location of the aneurysm.

### Tissue sampling and preparation

At the end of the surgery, a tissue fragment was removed from the remaining anterior wall of the AAA or any possible part of the PAA to be addressed without prejudice to the surgical technique dedicated to open aneurysm correction. This tissue fragment was excised, ensuring its longest dimension aligned with the vessel's longitudinal axis.

The removed fragment was immersed immediately in saline and transferred to the laboratory, where it was preserved at 4°C. Each fragment was analyzed ≤48 hours after collection. Before histological and biomechanical analyses, mural thrombus and atheroma plaques were removed from each tissue fragment collected during surgery through dissection of the aortic tissue.

After that, two specimens were produced using a device composed of two parallel blades, each with a length of 40 mm and a width of 5 mm. One was sent to a biomechanical test. The other was packaged in a 5% buffered formalin and later embedded in paraffin for the histological or immunohistochemical study identified with the same case number.^16^, ^17^, ^18^

### Uniaxial biomechanical test

The biomechanical analysis of arterial fragments followed established biomechanical testing principles for soft tissues.^19^^,^^20^

The Instron Inspec 2200 (Instron Corporation, Norwood, MA) and the AME 2 KN (Oswaldo Filizola, São Paulo, SP, Brazil) universal benchtop testers, equipped with a specimen bath and specialized soft tissue grips, were used for mechanical testing. Each rectangular tissue strip was secured at both ends by grips affixed to the crossheads of the respective tester.

**Fig 1:**
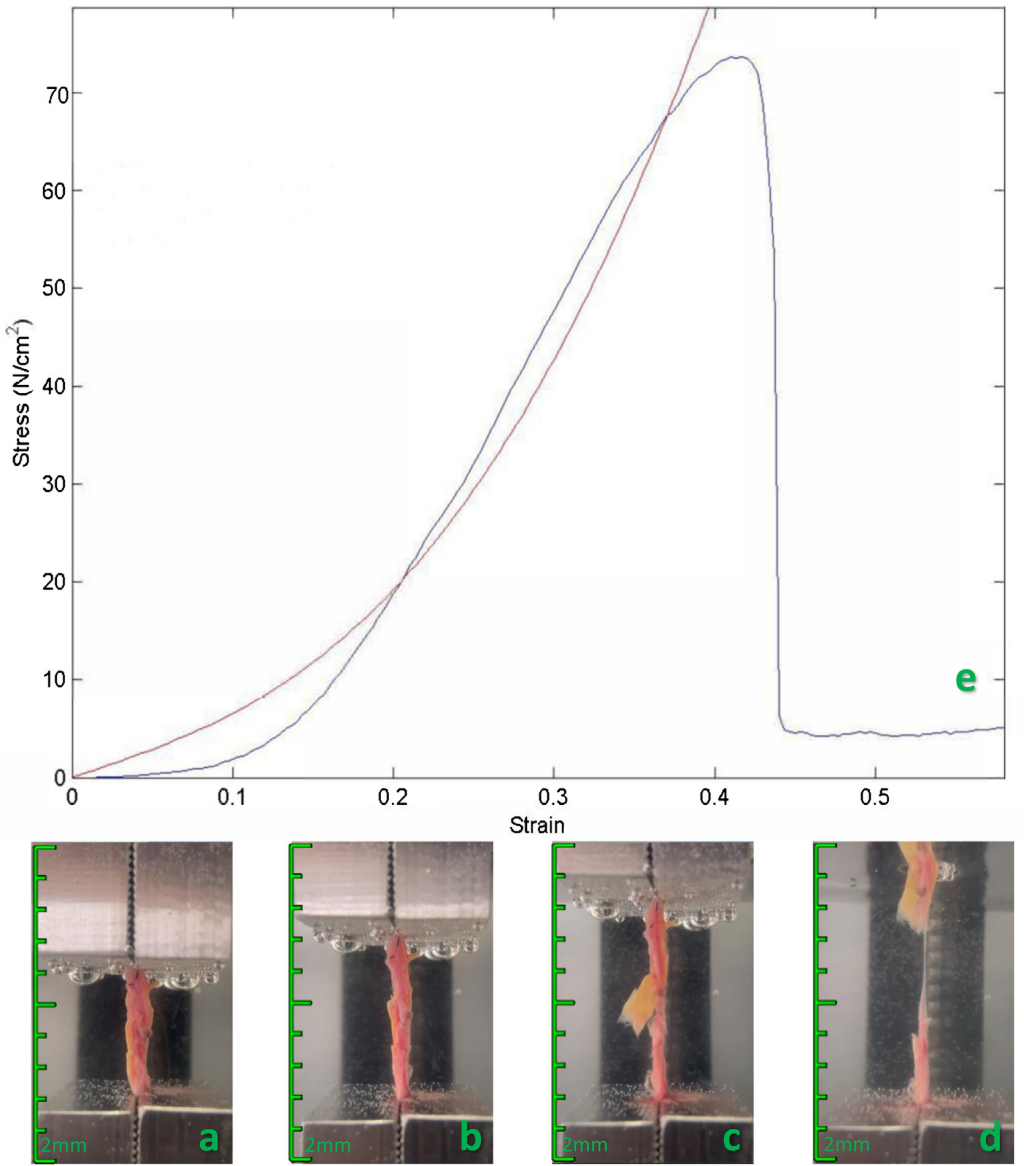
Biomechanical uniaxial tension test on arterial tissue. Schematic representation of the stages of a uniaxial tensile stress test and the corresponding stress-strain curve. **(A)** Test start. **(B)** Elastic and plastic phases. **(C)** Failure of the arterial fragment. **(D)** Rupture of the arterial fragment and test end. **(E)** Corresponding stress-strain curve. Green scale, each space on the scale represents 2 mm.

The initial length was determined by applying a negligible preload (0.01 N) to the specimen. Next, each specimen's length, width, and thickness between the clamps were measured and registered using a digital caliper. The width and thickness were measured at three locations and averaged. The specimen bath was filled with 0.9% saline solution at room temperature. First, a preconditioning test was performed involving 10 cyclic loading and unloading cycles of the specimen strip to 5% of its length at 20% of specimen length per minute. The preconditioning test aims to minimize the hysteresis effect observed in biological tissues, which is the dependence of the material's response on its loading history. The repeated loading and unloading cycles help to align the collagen fibers and reduce the energy dissipation associated with the rearrangement of these fibers during deformation. The low load used in the preconditioning protocol ensures that the tissue remains within its elastic limit, preventing any permanent damage or alteration of its mechanical properties. After preconditioning, the strip was uniaxially extended at 20% specimen length per minute until failure while recording force and elongation at an acquisition rate of 1 Hz. Fig 1 shows a schematic illustration of a uniaxial tension test on arterial tissue and its stress-strain curve.

The rupture site location and the characteristics were documented. The minor effect of upward thrust, the buoyant force exerted on the specimen and grips when submerged in the saline solution, was corrected by performing a control test without a specimen between the grips. The force-extension data recorded during the control test were then subtracted from the data obtained with the specimen to eliminate the influence of the buoyant force on the measurements.

### Biomechanical data analysis

In biomechanics, mechanical strength and elasticity refer to biological tissues' ability to withstand and recover from applied forces, like blood vessels.

Mechanical strength is the tissue's capacity to resist deformation or failure under various loads, such as tension (stretching), compression (squeezing), or shear (sliding forces). Determining how much stress a tissue can tolerate before rupturing or tearing is crucial.

Elasticity describes the tissue's ability to deform under stress and return to its original shape once removed. This property is essential for maintaining biological tissue's structural integrity and function. A highly elastic tissue can undergo significant deformation without permanent damage, whereas a less elastic tissue is more prone to injury.

The arterial wall fragments' greatest mechanical strength and elasticity were measured at the point of material failure. Material failure was defined as when the tissue could no longer withstand the applied stress or strain, resulting in rupture or excessive deformation during the uniaxial tensile test.^21^

The four mechanical strength parameters (also called resistance parameters^17^^,^^22^^,^^23^) measured at failure were failure load (also called maximum load) (F_f_), or the peak load applied to the specimen (in N); failure stress (S_f_), or the failure load per unit cross-sectional area of specimen strip (in N/cm^2^); failure tension (T_f_), or the failure load per unit width of specimen strip (in N/cm); and failure strain energy, or the energy accumulated by the specimen until the failure (in N/cm^2^).

The elasticity parameter measured at failure was failure strain (ε_f_), or the extension at failure as a fraction of the original length. The following equations were applied for the calculations:

(1) $$Sf=\left({Ff\;/\;w}_{0}t_{0}\right)\left(1+\varepsilon f\;\right)$$

(2) $$Tf=\left({Ff\;/\;w}_{0}\right)\left(\sqrt{1+\varepsilon f}\;\right)$$

(3) εf=(ι−ι0)/ι0,

where l_0_, w_0_, and t_0_ are the length, width, and thickness of the specimen strip between the grips at zero load, respectively; l is the length at the failure point; and ε_f_ accounts for the reduction in cross-sectional area in cases of stress calculations and reduction in width for tension calculations.^21^

A stress-strain curve was plotted for each sample tested. Strain energy was defined as the energy that was stored within a material when this material was deformed. Failure strain energy was calculated as the area under the curve of the stress-strain curve until the failure moment with the Curve Fitting software (MathWorks, Natick, MA).

### Subgroups based on aneurysm diameter

A subgroup analysis was conducted to investigate the influence of aneurysm diameter on its biomechanical properties. Aneurysms were stratified into two subgroups based on their maximum diameter: those <55 mm and ≥55 mm. The selection of the 55 mm cutpoint was based on AAA treatment guidelines and prior research on this topic.^1^^,^^2^^,^^16^

The analysis involved two critical comparisons: within each aneurysm type, smaller and larger aneurysms of the same type were compared to evaluate how size impacts biomechanics; between aneurysm types of similar size were compared to understand any biomechanical differences between the two aneurysm types, independent of size. This approach aimed to provide a clearer understanding of the relationship between aneurysm size and its biomechanical behavior.

### Subgroups based on AAA rupture

A statistical analysis of both groups was performed to evaluate the differences between the biomechanical variables of ruptured and nonruptured AAA. Owing to the limited sample size of histological and immunohistochemical variables in these subgroups, a comparative analysis of these characteristics was not feasible.

### Histological and immunochemistry analysis

Arterial tissue fragments were fixed in formalin and embedded in paraffin. Prior to histological staining, the paraffin sections were decalcified using EDTA.^24^^,^^25^ For histological analysis, 3-μm-thick sections were prepared and stained with hematoxylin and eosin to assess the thickness of the arterial layers, Verhoeff's stain to visualize and evaluate the elastic fibers within the tunica media, and Masson's trichrome stain to quantify the presence of collagen and fibrosis in all arterial layers.

Subsequent sections were subjected to immunohistochemistry for morphomolecular studies. For immunohistochemistry reactions, tissue sections three micrometers thick fixed on silanized slides were deparaffinized in three baths of xylol and rehydrated in three baths of 100% alcohol, two baths of 95% alcohol, and one bath of 70% alcohol. The recovery of antigenic sites was performed in Trilogy for 2 minutes of pressure at 121 psi in the PASCAL pan (DAKO Cytomation, Glostrup, Denmark). Endogenous peroxidase blocking was performed in two 20-minute baths with 6% hydrogen peroxide. Then, the sections were maintained for 30 minutes with CAS Block (Life Technologies, Carlsbad, CA) at room temperature to suppress the nonspecific protein binding of the reagents. The primary antibodies were maintained for 2 hours at 26°C and diluted in a specific reagent (Antibody Diluent Reagent Solution, Life Technologies). The Picture Max kit (Life Technologies) was the secondary antibody. The reaction was revealed with 3,3-diaminobenzidine tetrachloride (DAKO), resulting in a brown precipitate in places with the presence of antigen. The sections were stained with Harris' hematoxylin and then mounted with a coverslip using a suitable mounting medium.

Positive and negative controls underwent identical treatment procedures as the experimental samples, with the sole distinction being that negative controls were incubated exclusively with the antibody dilution solution, omitting the primary antibody. Incorporating positive and negative controls validated the specificity and accuracy of the antibodies used, thus ensuring the reliability of the immunohistochemical findings. These controls were excluded from the final data analysis.

The following immunohistochemical markers were investigated in each arterial layer: Alpha-actin (monoclonal mouse anti-human smooth muscle actin - clone 1A4, Code M0851, Dako Denmark A/S; dilution 1:150) was used to evaluate the percentage of smooth muscle cells within the tunica media. CD20 (monoclonal mouse anti-human CD20cy, clone L26, code M0755, Dako Denmark A/S; dilution 1:150) was used to assess the presence of B lymphocytes. CD45 (monoclonal mouse anti-human CD45RO, clone UCHL-1, code M072, Dako Denmark A/S; dilution 1:150) served as a pan-leukocyte marker. CD68 (monoclonal mouse anti-human CD68, clone KP1, code M0814, Dako Denmark A/S; dilution 1:500) was used to identify macrophages, monocytes, and Langerhans cells. PPARgamma (PPAR gamma polyclonal, rabbit/IgG, BS-4590R, Bioss Antibody, Carlsbad, CA; dilution 1:200) and KLF5 (anti-Human/Mouse KLF5 antibody, polyclonal goat IgG, catalog number AF3758, R&D Systems, Minneapolis, MN; dilution 1:200) were used to evaluate tissue adipogenic differentiation.^15^^,^^26^, ^27^, ^28^ MMP2 (MMP2 polyclonal antibody, rabbit/IgG, PA5-85,197, Invitrogen, Thermo Fisher Scientific, Marlborough, MA; dilution 1:500) was a marker of extracellular matrix degradation.

After histological and immunohistochemical processing, the slides were digitized using the Scanscope CS System (Aperio Technology Solutions, Inc., Boston, MA), equipped with an Olympus UPlanSApo 20x objective lens. These high-resolution digital images (.svs files) were then analyzed using Aperio ImageScope View Software, obviating the need for a conventional optical microscope. After a trained biologist configured the software parameters, automated analyses were conducted to determine the total positive percentage and number of positive cells in the selected area per square millimeter. Additionally, the software was used to measure the thickness of the intimal and adventitia layers and quantify the percentage of elastic fibers in the tunica media and the percentage of collagen/fibrosis across all arterial layers.

### Statistical analysis

Categorical variables were presented as absolute (n) and relative (%) frequencies. Quantitative variables were described using means, standard deviations, and medians with interquartile ranges (first and third quartiles).

Parametric tests were used when the assessment of distribution plots and the Shapiro-Wilk test indicated normality in the data distribution; in these cases, variables were compared using the Student *t* test, with Welch's correction applied if Brown-Forsythe's test indicated unequal variances. Nonparametric tests were used when the assessment of distribution plots and the Shapiro-Wilk test did not indicate normality in the data distribution; in these instances, the Mann-Whitney *U* test was used.

Statistical significance was set at a *P* value of <.05. All analyses were conducted using SPSS software version 24 (IBM, Armonk, NY) and JASP version 0.18.3 (2024, University of Amsterdam).

## Results

### General results and tables

**Fig 2:**
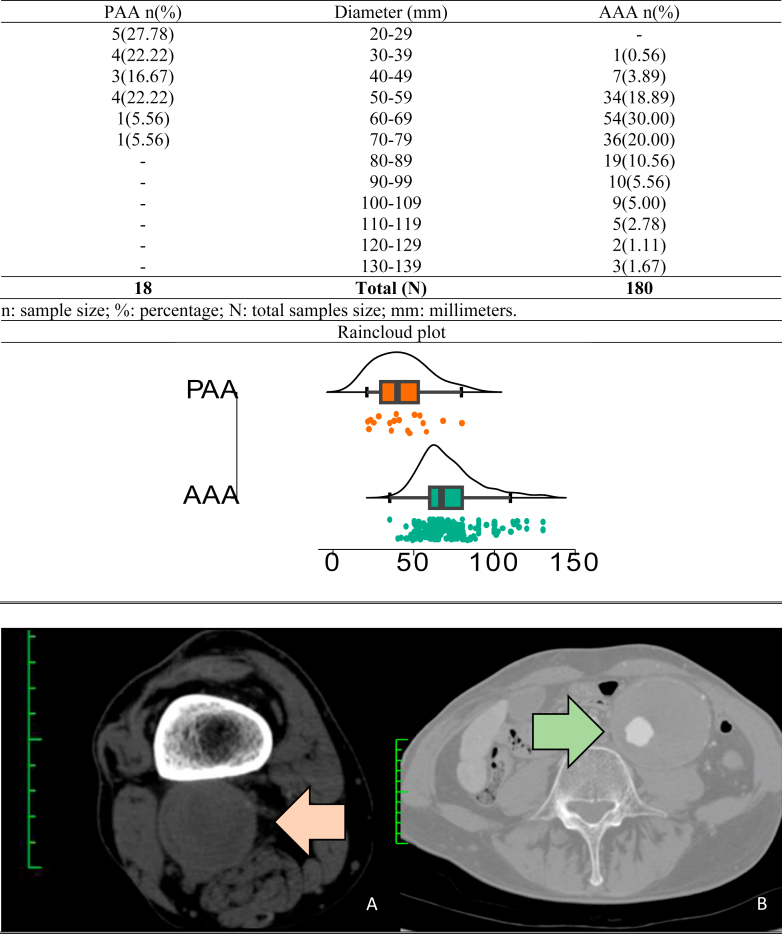
Table detailing the number of individuals within each aneurysm sac size length interval, accompanied by a distribution plot and representative angiotomography images for each aneurysm group. Abdominal aortic aneurysm (*AAA*) and popliteal artery aneurysm (*PAA*). The *x*-axis represents the diameter size in millimeters, and the *y*-axis indicates the density of samples exhibiting that specific diameter. Individual data points are represented by small dots. Representative angiotomography images of the AAA (**A**, *orange arrow*) and the PAA (**B**, *green arrow*). Green scales, each space on the scales represents 10 mm.

A total of 180 AAA and 18 PAA specimens, along with their associated data, were collected for this study. Descriptive statistics for the AAA group are presented in Supplementary Table I, and those for the PAA group are detailed in Supplementary Table II. Fig 2 presents a table detailing the number of individuals within each aneurysm sac size length interval, accompanied by a distribution plot and representative angiotomography images for each aneurysm group.

The smallest AAA measured 35 mm, and the smallest PAA was 21.22 mm. All AAAs <50 mm (9 cases [5%]) were collected during emergency surgeries for ruptured AAA.

Aneurysm sac diameter was the primary indication for surgery in both the AAA and PAA groups. However, other factors also contributed to surgical intervention. In the AAA group, 35 (19.4%) cases required surgery owing to AAA rupture. In contrast, within the PAA group, 5 cases (27.7%) underwent surgery owing to critical limb ischemia caused by PAA thrombosis, and 1 case (5.5%) was due to distal limb embolization originating from the PAA. Importantly, no surgeries were performed owing to PAA rupture in this study.

### Subgroup comparisons

The comparative analysis of AAA subgroups based on diameter (<55 mm and ≥55 mm) is presented in Table I. The AAA ≥55 mm subgroup exhibited significantly thicker arterial walls (1.9 mm vs 1.6 mm; *P* = .025) and greater mechanical strength compared with the AAA <55 mm subgroup, as evidenced by a higher failure load (7.3 N vs 5.1 N; *P* = .004) and higher failure tension (13.6 N/cm vs 11.1 N/cm; *P* = .004). No significant differences were observed in biomechanical variables between the PAA <55 mm and PAA ≥55 mm subgroups.

**Table I:** Comparative analysis of abdominal aortic aneurysm (AAA) subgroups based on diameter (<55 mm and ≥55 mm)

	AAA <55 mm			AAA ≥55 mm			*P* value
	No.	Median	IQR	No.	Median	IQR	
Clinical variables							
Diameter, mm	26	50.5	(48-53)	154	70.5	(63-80.8)	**.001**
Thickness, mm	26	1.6	(1.4-2.0)	154	1.9	(1.48-2.3)	**.025**
Biomechanical variables (at failure)							
Load (N)	26	4.4	(2.5-5.6)	154	5.39	(4.1-7.1)	**.004**
Stress (N/cm^2^)	26	74.4	(56.7-92.0)	154	89.3	(66.3-115.6)	.054
Tension (N/cm)	26	11.1	(6.8-14.1)	154	13.6	(10.2-18.2)	**.003**
Strain energy (N/cm^2^)	6	10.8	(10.5-11.1)	94	9.8	(7.4-13.6)	.902
Strain %	25	0.4	(0.4-0.5)	154	0.3	(0.3-0.4)	.140

*IQR,* Interquartile range.

Boldface entries indicate statistical significance. The statistical test used in all table analyses was the Mann-Whitney *U* test.

When comparing the AAA <55 mm subgroup with the PAA <55 mm subgroup (Table II), the PAA <55 mm subgroup demonstrated greater mechanical strength, characterized by a higher failure load (6.4 N vs 4.3 N; *P* = .028) and higher failure tension (16.9 N/cm vs 11.2 N/cm; *P* = .032). No differences were observed between the AAA ≥55 mm and PAA ≥55 mm subgroups.

**Table II:** Comparative analysis between abdominal aortic aneurysm (*AAA*) <55 mm and popliteal artery aneurysm (*PAA*) <55 mm subgroups

	AAA <55 mm		PAA <55 mm		*P* value
	No.		No.		
Clinical variables					
Diameter, mm^b^	26	50.5 (48-53)	14	36.8 (25.9-44.7)	**.001**
Thickness, mm^a^	26	1.7± 0.5	14	1.9 ± 0.6	.171
Biomechanical variables (at failure)					
Load (N)^a^	26	4.3 ± 2.5	14	6.4 ± 3.4	**.028**
Stress (N/cm^2^)^b^	26	74.4 (56.7-92)	14	93.3 (70.4-137.9)	.156
Tension (N/cm)^a^	26	11.2 (6.6)	14	16.9 (9.6)	**.032**
Strain energy (N/cm^2^)^b^	6	10.8 (15.8-11.1)	14	13.4 (10-19.8)	.116
Strain %^b^	25	0.4 (0.4-0.5)	14	0.3 (0.3-0.6)	.671

Values are median (interquartile range) or mean ± standard deviation.

Boldface entries indicate statistical significance.

a Student *t* test.

b Mann-Whitney *U* test.

The comparison between biomechanical variables of ruptured and nonruptured AAA revealed no statistically significant differences between the two groups. This subgroup comparison is presented in Table III.

**Table III:** Comparative analysis between ruptured abdominal aortic aneurysm (*AAA*) and nonruptured AAA subgroups

	Ruptured AAA		Nonruptured AAA		*P* value
	No.		No.		
Clinical variables					
Diameter, mm	35	75 (56-95)	145	65 (60-77.4)	.071
Thickness, mm	35	1.8 (1.5-2.0)	145	1.9 (1.4-2.2)	.468
Biomechanical variables (at failure)					
Load (N)	35	4.8 (3.2-6.5)	145	5.2 (4.1-6.9)	.304
Stress (N/cm^2^)	35	78.1 (60.5-124.1)	145	87.8 (67.4-113.6)	.598
Tension (N/cm)	35	12.3 (8.4-16.7)	145	13.3 (10.1-18.0)	.237
Strain energy (N/cm^2^)	20	11.6 (8.1-13.8)	80	9.7 (7.2-12.9)	.799
Strain %	35	0.4 (0.3-0.4)	144	0.4 (0.3-0.4)	.278

Owing to the small sample size within each subgroup, histological and immunohistochemical variables were not compared between subgroups.

Values are median (interquartile range) unless otherwise indicated. Boldface entries indicate statistical significance. The statistical test used in all table analyses was the Mann-Whitney *U* test.

### AAA vs PAA group comparisons

A comparative analysis of the AAA and PAA groups is summarized in Table IV. Biomechanical evaluation demonstrated that PAA walls exhibited higher mechanical strength than AAA walls, as evidenced by a significantly higher failure strain energy (13.36 N/m^2^ vs 9.95 N/m^2^; *P* = .023). No significant difference in elasticity was observed between the two groups.

**Table IV:** Comparative analysis between abdominal aortic aneurysm (*AAA*) and popliteal artery aneurysm (*PAA*) groups

	AAA		PAA		*P* value
	No.		No.		
Clinical variables					
Age, years^d^	180	69.50 (65-75)	18	68.50 (66.25-78.50)	.424
Diameter, mm2	180	67 60-80		39.80 29.83-52.78	**<.001**^b^
Thickness, mm2	180	1.83 (1.45-2.21)	18	1.845 (1.45-2.24)	.811
Biomechanical variables (at failure)					
Load (N)^d^	180	5.11 (3.78-6.78)	18	6.15 (4.07-8.40)	.389
Stress (N/cm^2^)^d^	180	86.24 (64.46-115.48)	18	108.41 (70.42-158.37)	.195
Tension (N/cm)^d^	180	13.18 (9.98-17.96)	18	15.78 (10.30-20.76)	.406
Strain energy (N/cm^2^)^d^	100	9.95 (7.41-13.46)	18	13.36 (10.00-20.23)	**.023**^a^
Strain, %^d^	179	0.36 (0.31-0.44)	18	0.31 (0.27-0.56)	.783
Histological variables					
HE - intima layer - thickness, mm^d^	23	0.50 (0.26-0.93)	15	0.41 (0.25-1.07)	.988
HE – Adventitia layer - Thickness, mm^c^	21	1.46 ± 0.88	15	0.98 ± 0.57	.075
Verhoeff - tunica media – elastic fibers %^c^	21	38.05 ± 16.30	13	41.69 ± 21.79	.582
Masson – all layers - fibrosis %^c^	21	49.43 ± 15.77	15	40.53 ± 14.11	.091
Imunohistochemical variables					
Actina - tunica media %^c^	15	22.69 ± 14.29	10	21.22 ± 12.10	.791
CD20 - intima layer - PC^d^	14	11 (3.75-44.75)	15	20 (8-50)	.419
CD20 - tunica media - PC^d^	14	5 (3.25-18.25)	15	5 (3.5-32)	.583
CD20 - adventitia layer - PC^d^	14	1475.50 (1031.75-2086)	15	320 (79.50-754.50)	**.003**^a^
CD45 - intima layer - PC^d^	14	29 (7-69.75)	15	23 (7-68)	.930
CD45 - tunica media - PC^d^	14	21.50 (5.25-110.75)	15	24 (8-57.5)	.913
CD45 - adventitia layer - PC^d^	14	1731.50 (683.25-2830)	15	409 (252-2010.50)	.172
CD68 - intima layer - PC^d^	15	200 (19-761.50)	14	38 (18.50-405.25)	.458
CD68 - tunica media - PC^d^	15	53 (17-425.50)	14	21 (2.75-67.25)	.175
CD68 - adventitia layer - PC^d^	15	1533 (105.5-2373)	14	374 (118-1035.75)	.310
PPARgamma - intima layer - PC^d^	14	714.50 (206-2377)	14	230 (47.25-1800)	.223
PPARgamma - tunica media - PC^d^	14	291.50 (166.25-927.75)	14	85.50 (23.25-481.50)	.124
PPARgamma - adventitia layer - PC^d^	14	4854.50 (2510-8092.75)	14	778 (167.25-3787.25)	**.009**^a^
KLF5 - intima layer - PC^d^	14	77.50 (44.25-164.50)	14	283.50 (124.25-508.50)	**.039**^a^
KLF5 - tunica media - PC^d^	14	11 (8-25.75)	14	26 (9.25-47.75)	.312
KLF5 - adventitia layer - PC^d^	14	1082 (501.25-1544.50)	14	1013.50 (606.25-2514.50)	.306
MMP2 - intima layer - PC^d^	14	121.50 (87.75-457.25)	14	385 (174.50-1053)	.175
MMP2- tunica media - PC^d^	14	95.50 (46-257.75)	14	99 (34.25-181)	.800
MMP2- adventitia layer - PC^d^	14	2683.50 (2081.25-3156.25)	14	2517 (1316.25-3614)	.946

*MMP,* Matrix metalloproteinase; *PC,* positive cells count per square millimeter.

Values are mean ± standard deviation or median (interquartile range) unless otherwise noted. Boldface entries indicate statistical significance.

a *P* < .05.

b *P* < .001.

c Student *t* test.

d Mann-Whitney *U* test.

**Fig 3:**
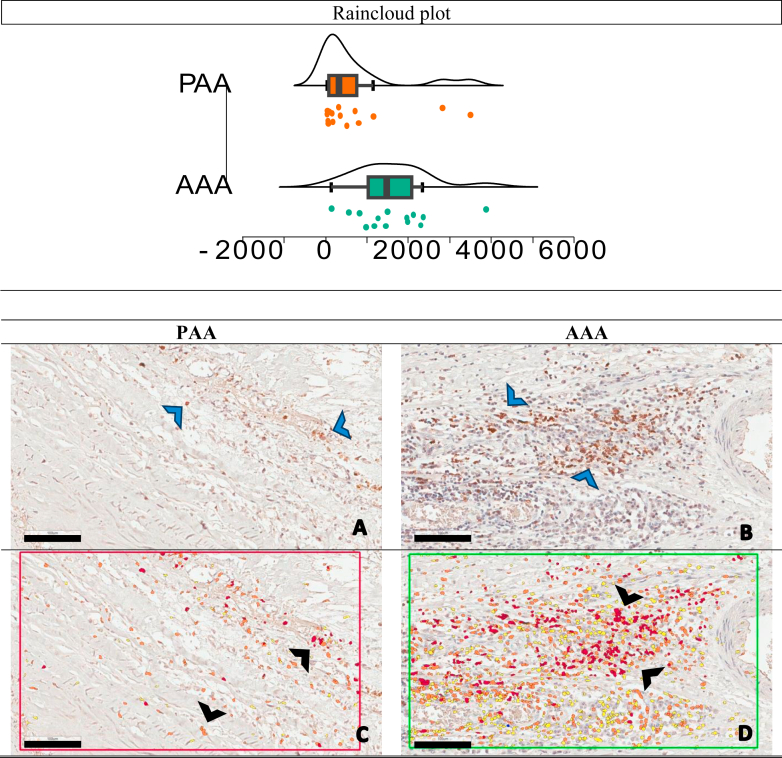
CD20-positive cell counts in the adventitia layer: abdominal aortic aneurysm (*AAA*) vs popliteal artery aneurysm (*PAA*), distribution plots and representative immunohistochemical staining. Raincloud plot. The *x*-axis represents the positive cells count per square millimeter, and the *y*-axis indicates the density of samples with that cell count. The small dots represent individual data points. A statistically significant difference in CD20 expression was observed between AAA **(B** and **D)** and PAA **(A** and **C)** samples (*P* = .003), with CD20-positive cells significantly more abundant in AAA adventitia layer. The figure displays four micrographs illustrating the adventitial layer of AAA and PAA samples, stained for CD20, a marker of B lymphocytes. Positive immunodetection is visualized as brown staining (*blue arrows*), indicating the presence of CD20 **(A** and **B)**. To quantify CD20 expression, Aperio Software was used to automatically identify and color-code CD20-positive cells in red (*black arrows*), enabling precise cell counting per square millimeter **(C** and **D)**. *Scale bars*, 100 μm (magnification ×20), provide a reference for image size, facilitating comparison of staining intensity and cellular distribution.

Furthermore, histological analysis revealed no statistically significant differences in any of the assessed variables between the PAA and AAA groups. Immunohistochemical analysis, however, demonstrated a significantly higher count of B lymphocyte cells (CD20) in the adventitia layer of the AAA compared with the PAA (1475.50 vs 320; *P* = .003), as illustrated in Fig 3.

**Fig 4:**
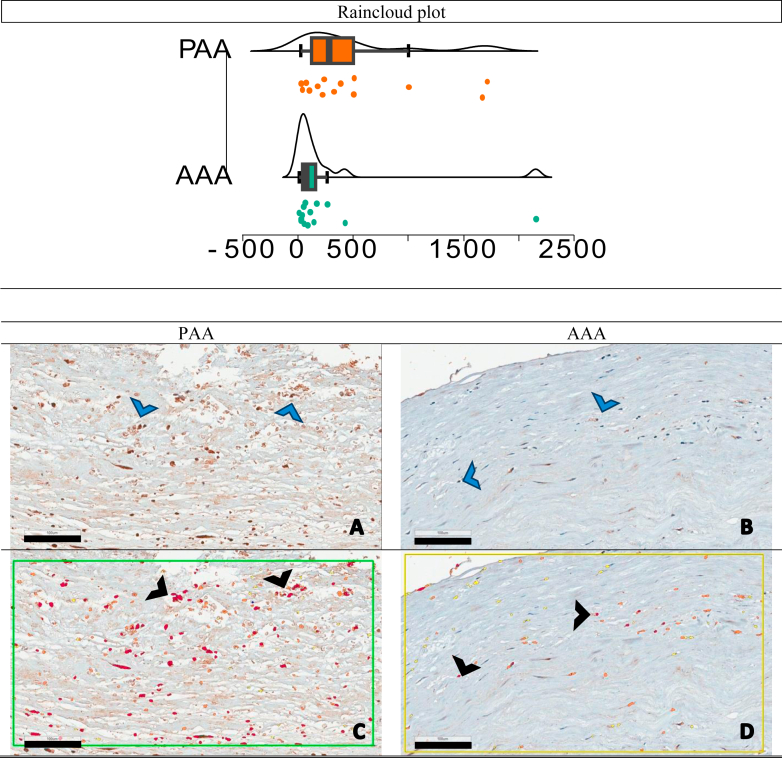
KLF5-positive cell counts in the intima layer: abdominal aortic aneurysm (*AAA*) vs popliteal artery aneurysm (*PAA*), distribution plots and representative immunohistochemical staining. The *x*-axis represents the positive cells count per square millimeter, and the *y*-axis indicates the density of samples with that cell count. The small dots represent individual data points. A statistically significant difference in KLF5 expression was observed between AAA **(B** and **D)** and PAA **(A** and **C)** samples (*P* = .039). KLF5-positive cells were significantly more abundant in PAA intima layer. The figure displays four micrographs illustrating the intimal layer of AAA and PAA samples, stained for KLF5, a marker of adipogenic differentiation. Positive immunodetection is visualized as brown staining (*blue arrows*), indicating the presence of KLF5 **(A** and **B)**. To quantify KLF5 expression, Aperio Software was used to automatically identify and color-code KLF5-positive cells in red (*black arrows*), enabling precise cell counting per square millimeter **(C** and **D)**. *Scale bars*, 100 μm (magnification ×20), provide a reference for image size, facilitating comparison of staining intensity and cellular distribution.

**Fig 5:**
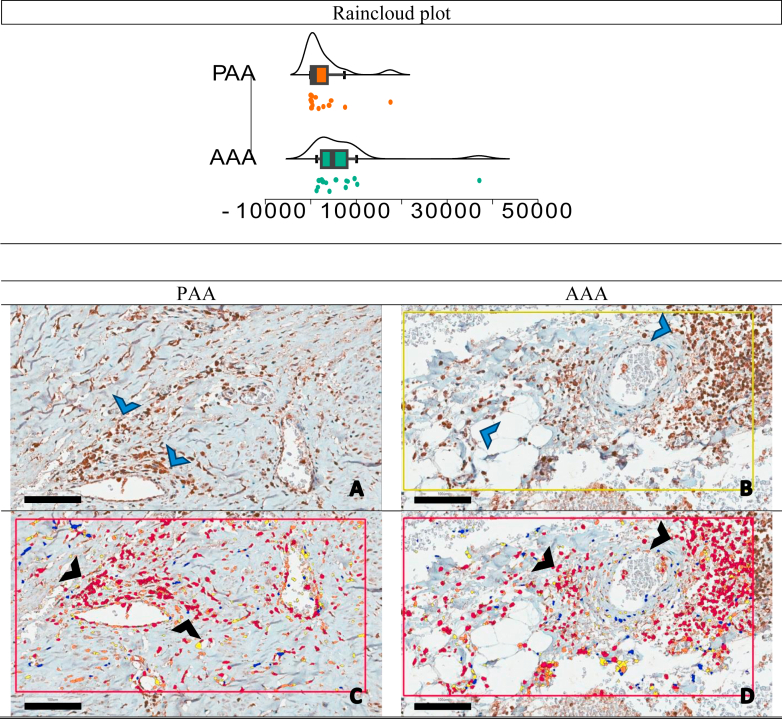
PPARgamma-positive cell counts in the adventitia Layer: abdominal aortic aneurysm (*AAA*) vs popliteal artery aneurysm (*PAA*), distribution plots and representative immunohistochemical staining. The *x*-axis represents the positive cells count per square millimeter, and the *y*-axis indicates the density of samples with that cell count. The small dots represent individual data points. A statistically significant difference in PPARgamma expression was observed between AAA and PAA samples (*P* = .009). PPARgama-positive cells were significantly more abundant in AAA adventitia layer. The figure displays four micrographs illustrating the adventitial layer of AAA and PAA samples, stained for PPARgamma, a marker of adipogenic differentiation. Positive immunodetection is visualized as brown staining (*blue arrows*), indicating the presence of PPARgamma **(A** and **B)**. To quantify PPARgamma expression, Aperio Software was used to automatically identify and color-code PPARgama-positive cells in red (*black arrows*), enabling precise cell counting per square millimeter **(C** and **D)**. *Scale bars*, 100 μm (magnification ×20), provide a reference for image size, facilitating comparison of staining intensity and cellular distribution.

Additionally, the analysis revealed increased KLF5 expression within the intimal layer of the PAA compared with the AAA (283.50 vs 77.50; *P* = .039) (Fig 4). In contrast, PPARgamma expression was notably higher in the AAA's adventitia layer than the PAA (4854.50 vs 778; *P* = .009, Fig 5).

## Discussion

The present study identified that PAA wall fragments exhibited greater mechanical strength than AAA. At the same time, AAA exhibited an increased presence of B lymphocytes within the adventitia, and distinct patterns of adipogenic differentiation, with prominence in the PAA intima and AAA adventitia. The findings underscored the divergent pathophysiological processes involved in AAA and PAA development and progression; PAA walls exhibited significantly greater mechanical strength than AAA walls, even with significantly smaller diameters. The mechanical strength discrepancy suggests AAA's inherent vulnerability to structural failure under pressure. However, it raises the question of whether this difference in mechanical strength is attributable to diameter or aneurysm type.

The literature lacks direct comparative biomechanical studies between AAAs and PAAs. Without articles directly comparing these two types of aneurysms, one approach is to examine biomechanical studies on AAAs that compare aneurysms ≥55 mm and <55 mm in diameter. One study demonstrated higher mechanical strength in the AAA ≥55 mm subgroup than in AAA <55 mm, represented by greater failure load, failure stress, and failure tension.^17^ If bigger aneurysms inherently possess greater mechanical strength, then the smaller size of the PAA group should not account for their superior mechanical strength compared with the AAA group.

Subgroup analyses were performed based on maximum aneurysm diameter to answer this question. It was found that the AAA ≥55 mm subgroup exhibited greater mechanical strength (indicated by higher failure load and higher failure tension) compared with AAA <55 mm, confirming the findings of the study mentioned above. A comparison was performed between both aneurysm subgroups with <55 mm in diameter to try to decrease the influence of diameter in the analysis. Notably, the PAA group still had smaller diameters than the AAA group. The PAA <55 mm subgroup demonstrated higher mechanical strength (indicated by a greater failure load and higher failure tension) when compared with AAA <55 mm. These results support the hypothesis that aneurysm type is a critical factor in explaining the lower mechanical strength in the AAA group.

No differences in biomechanical variables were noted between different sizes of PAA (<55 mm and ≥55 mm) or between AAA ≥55 mm and PAA ≥55 mm. One of the main reasons for this was likely the small sample size of the PAA ≥55 mm group (four specimens).

Additionally, to analyze the complexity of aneurysms, there was no difference in biomechanical variables between subgroups of ruptured AAAs and nonruptured AAAs. This finding corroborates data from the same previous article and may be explained by the heterogeneity of the aneurysm wall formation and growth processes.^17^

Immunohistochemical comparison of AAA and PAA walls reveals higher count of B lymphocyte cells (CD20) in the adventitia layer of the AAA, where adipogenic differentiation is also more prominent compared with PAA. In contrast, PAA demonstrates a more significant adipogenic differentiation (KLF5) in the intima layer when compared with AAA.

These findings are consistent with the existing literature, demonstrating distinct inflammatory patterns and adipogenic differentiation between AAAs and PAAs. Although both aneurysm types exhibit these processes across all layers, AAAs show increased severity in the outermost layer (adventitia) when compared with PAAs, and PAAs show increased severity in the innermost layer (intima) when compared with AAAs.^14^^,^^15^^,^^29^

Furthermore, in both aneurysms, the adventitia is the most affected layer by inflammatory, apoptotic, and adipogenic degenerative processes compared with the other layers. Although these processes also occur in the inner layers, they are less pronounced. These observations support the outside-in theory of aneurysm formation, suggesting that the adventitia is the primary layer affected by the aneurysmal disease.^14^^,^^17^^,^^30^, ^31^, ^32^, ^33^

### Limitations

Limitations of the current study include the inability to conduct histological and immunohistochemical analyses based on subgroups. Expanding the sample size could address some of these questions. However, the increasing prevalence of endovascular surgery has reduced the availability of arterial specimens (both aneurysmal and nonaneurysmal) for research into the pathophysiology of aneurysm formation and complications.

The asymmetry in group sizes, differing sample sizes, and the nonrandomized group formation prevented statistical comparisons of clinical variables between the groups. The extended duration of AAA sample collection (2004 to 2021) may have introduced more significant variability owing to potential changes in surgical techniques or patient demographics. The AAA group's more significant heterogeneity in terms of sex could also contribute to the observed variance in biomechanical properties. Additionally, variations in comorbidities and clinical variables, such as weight and medication use, although not explicitly investigated in this study, might further contribute to the heterogeneity within the AAA group.

During AAA sample collection, obtaining fragments in nonlongitudinal axes (eg, circumferential or radial) often posed a risk of disrupting the surgical technique of endoaneurysmorrhaphy, where the aneurysm sac is carefully sutured over the Dacron graft to safeguard the bowel. Although investigating biomechanical properties in these alternative axes could offer valuable insights, prioritizing patient safety and minimizing potential complications during surgery guided the decision to focus primarily on longitudinal sampling. In addition, obtaining and preparing samples for circumferential or radial testing from smaller aneurysms like PAA presents significant technical challenges.

Although the assumptions of homogeneity and isotropy in the arterial wall simplify the biomechanical analysis, they may not capture fully the complex, heterogeneous nature of aneurysmal tissue. The observed measurement variances could be attributed partly to the arterial wall's intrinsic inhomogeneity and anisotropy, which exhibits regional and interindividual variations in composition, structure, and mechanical properties.

## Conclusions

PAA wall fragments demonstrate greater mechanical strength compared with AAA wall fragments. In contrast, AAA walls contain a higher number of B lymphocytes within the adventitia compared with PAA walls. Adipogenic differentiation is more pronounced in the adventitia of AAA than in PAA, while in PAA, it is more prominent in the intima compared with AAA.

## Declaration of Generative AI and AI-Assisted technologies in the writing process

During the preparation of this work, the authors used Gemini Advanced (Alphabet, 2024) and Grammarly Premium (Grammarly, 2024) for English language translation and to adhere to the *Journal of Vascular Surgery*–Vascular Science style guide. After using this tool/service, the authors reviewed and edited the content as needed and took full responsibility for the publication's content.

## Author Contributions

Conception and design: MO, AB, ES

Analysis and interpretation: MO, AB, ES

Data collection: MO, AB, GS, AS, MM, MR, MH, ES

Writing the article: MO, AB, ES

Critical revision of the article: MO, AB, GS, AS, MM, MR, MH, ES

Final approval of the article: MO, AB, GS, AS, MM, MR, MH, ES

Statistical analysis: MO

Obtained funding: MO, AB, ES

Overall responsibility: MO

## Funding

This work was funded by the 10.13039/501100001807São Paulo Research Foundation (Fundação de Amparo à Pesquisa do Estado de São Paulo, FAPESP), a public foundation in São Paulo, Brazil, through protocol #2021/02,193-7. The funder was not involved in the study's design, data collection, analysis, or reporting.

## Disclosures

None.
