# Supplementary material for: Comparative analysis between abdominal aortic aneurysm and popliteal artery aneurysm
Source: JVS Vasc Sci. 2024 Dec 28;6:100279. doi: 10.1016/j.jvssci.2024.100279 (PMC11815950; doi:10.1016/j.jvssci.2024.100279)
Supplement: Supplementary material [file mmc1.docx]

Supplemental Table 1. Descriptive findings of the AAA group.

|  | AAA | | | | | | |
| --- | --- | --- | --- | --- | --- | --- | --- |
|  | n | Mean | SD | Min | Max | Median | IQR |
| **CLINICAL VARIABLES** |  |  |  |  |  |  |  |
| Male n (%) | 130(72.22%) |  |  |  |  |  |  |
| Smoking n (%) | 151(84.36%) |  |  |  |  |  |  |
| SAH n (%) | 148(82.68%) |  |  |  |  |  |  |
| DM n (%) | 22(16.29%) |  |  |  |  |  |  |
| Age (years) | 180 | 70.10 | 6.15 | 60 | 85 | 69.50 | 65.00-75.00 |
| Diameter (mm) | 180 | 71.43 | 18.02 | 35 | 130 | 67.00 | 60.00-80.00 |
| Wall Thickness (mm) | 180 | 1.91 | 0.59 | 0.79 | 4.00 | 1.83 | 1.45-2.21 |
| **BIOMECHANICAL VARIABLES**  **(AT FAILURE)** |  |  |  |  |  |  |  |
| Load (N) | 180 | 5.87 | 3.31 | 0.07 | 21.90 | 5.11 | 3.78-6.78 |
| Stress (N/cm2) | 180 | 94.91 | 43.49 | 1.02 | 246.48 | 86.24 | 64.46-115.48 |
| Tension (N/cm) | 180 | 15.20 | 8.87 | 0.15 | 63.98 | 13.19 | 9.98-17.96 |
| Strain energy (N/cm^2^) | 100 | 12.43 | 9.65 | 1.69 | 56.78 | 9.95 | 7.41-13.46 |
| Strain % | 179 | 0.38 | 0.12 | 0.05 | 0.85 | 0.36 | 0.31-0.44 |
| **HISTOLOGICAL VARIABLES** |  |  |  |  |  |  |  |
| HE - Intima layer – Thickness (mm) | 23 | 0.64 | 0.43 | 0.10 | 1.50 | 0.50 | 0.26-0.94 |
| HE – Adventitia layer - Thickness (mm) | 21 | 1.46 | 0.88 | 0.10 | 3.60 | 1.20 | 0.90-2.20 |
| Verhoeff – Media – Elastic Fibers % | 21 | 38.05 | 16.30 | 0.00 | 74.00 | 38.00 | 30.00-43.00 |
| Masson – All layers – Fibrosis % | 21 | 49.43 | 15.77 | 28.00 | 83.00 | 46.00 | 36.00-61.00 |
| **IMUNOHISTOCHEMICAL VARIABLES** |  |  |  |  |  |  |  |
| Actina - Tunica media % | 15 | 22.69 | 14.29 | 5.40 | 48.00 | 19.00 | 12.00-34.00 |
| CD20 - Intima layer - PC | 14 | 55.71 | 121.36 | 0.00 | 465.00 | 11.00 | 3.75-44.75 |
| CD20 - Tunica media - PC | 14 | 11.71 | 14.13 | 1.00 | 50.00 | 5.00 | 3.25-18.25 |
| CD20 - Adventitia layer - PC | 14 | 1608.21 | 930.12 | 151.00 | 3871.00 | 1475.50 | 1031.75-2086.00 |
| CD45 - Intima layer - PC | 14 | 133.500 | 366.42 | 3.00 | 1401.00 | 29.00 | 7.00-69.75 |
| CD45 - Tunica media - PC | 14 | 62.36 | 73.03 | 2.00 | 190.00 | 21.50 | 5.25-110.75 |
| CD45 - Adventitia layer - PC | 14 | 2521.10 | 2558.48 | 16.40 | 8204.00 | 1731.50 | 683.25-2830.00 |
| CD68 - Intima layer - PC | 15 | 754.07 | 1141.18 | 1.00 | 3172.00 | 200.00 | 19.000-761.50 |
| CD68 - Tunica media - PC | 15 | 257.87 | 334.57 | 1.00 | 885.00 | 53.00 | 17.00-425.50 |
| CD68 - Adventitia layer - PC | 15 | 1346.73 | 1323.49 | 14.00 | 3662.00 | 1533.00 | 105.50-2373.00 |
| PPARgama - Intima layer - PC | 14 | 1846.71 | 2999.60 | 9.00 | 11572.00 | 714.50 | 206.00-2377.00 |
| PPARgama - Tunica media - PC | 14 | 665.00 | 800.16 | 6.00 | 2682.00 | 291.50 | 166.25-927.75 |
| PPARgama - Adventitia layer - PC | 14 | 7413.36 | 9087.53 | 1346.00 | 37096.00 | 4854.50 | 2510.00-8092.75 |
| KLF5 - Intima layer - PC | 14 | 261.21 | 557.85 | 12.00 | 2159.00 | 77.50 | 44.25-164.50 |
| KLF5 - Tunica media - PC | 14 | 29.64 | 47.32 | 1.00 | 182.00 | 11.00 | 8.00-25.75 |
| KLF5 - Adventitia layer - PC | 14 | 1225.36 | 1079.99 | 78.00 | 4094.00 | 1082.00 | 501.25-1544.50 |
| MMP2 - Intima layer - PC | 14 | 638.93 | 1455.69 | 18.00 | 5624.00 | 121.50 | 87.75-457.25 |
| MMP2- Tunica media - PC | 14 | 157.64 | 144.73 | 6.00 | 436.00 | 46.00 | 95.50-257.75 |
| MMP2- Adventitia layer - PC | 14 | 2841.07 | 1696.12 | 880.00 | 7599.00 | 2683.50 | 2081.25-3156.25 |

n: sample size; %: percentage; mm: millimeters; SD: standard deviation; PC: positive cells count per mm^2^; Min: minimum value; Max: maximum value; IQR: interquartile range (Q1-Q3); SAH: Systemic Arterial Hypertension; DM: Diabetes Mellitus.
