# Supplementary material for: Comparative analysis between abdominal aortic aneurysm and popliteal artery aneurysm
Source: JVS Vasc Sci. 2024 Dec 28;6:100279. doi: 10.1016/j.jvssci.2024.100279 (PMC11815950; doi:10.1016/j.jvssci.2024.100279)
Supplement: Supplementary material [file mmc2.docx]

Supplemental Table 2. Descriptive findings of the PAA group.

|  | PAA | | | | | | |
| --- | --- | --- | --- | --- | --- | --- | --- |
|  | n | Mean | SD | Min | Max | Median | IQR |
| **CLINICAL VARIABLES** |  |  |  |  |  |  |  |
| Male n (%) | 18(100) |  |  |  |  |  |  |
| Smoking n (%) | 7(38.89) |  |  |  |  |  |  |
| SAH n (%) | 17(94.44) |  |  |  |  |  |  |
| DM n (%) | 7(38.89) |  |  |  |  |  |  |
| Age (years) | 18 | 71.67 | 7.19 | 62.00 | 83.00 | 68.50 | 66.25-78.50 |
| Diameter (mm) | 18 | 42.54 | 16.40 | 21.22 | 79.80 | 39.80 | 29.83-52.78 |
| Wall Thickness (mm) | 18 | 1.84 | 0.59 | 0.87 | 3.15 | 1.84 | 1.45-2.24 |
| **BIOMECHANICAL VARIABLES**  **(AT FAILURE)** |  |  |  |  |  |  |  |
| Load (N) | 18 | 6.38 | 3.23 | 1.90 | 11.70 | 6.15 | 4.07-8.40 |
| Stress (N/cm2) | 18 | 113.40 | 59.12 | 32.19 | 215.66 | 108.41 | 70.42-158.38 |
| Tension (N/cm) | 18 | 16.69 | 8.90 | 4.69 | 33.03 | 15.78 | 10.30-20.77 |
| Strain energy (N/cm^2^) | 18 | 16.97 | 12.92 | 2.18 | 57.94 | 13.36 | 10.00-20.23 |
| Strain % | 18 | 0.41 | 0.19 | 0.22 | 0.77 | 0.32 | 0.27-0.56 |
| **HISTOLOGICAL VARIABLES** |  |  |  |  |  |  |  |
| HE - Intima layer – Thickness (mm) | 15 | 0.72 | 0.66 | 0.11 | 2.20 | 0.41 | 0.25-1.07 |
| HE – Adventitia layer - Thickness (mm) | 15 | 0.98 | 0.57 | 0.34 | 2.20 | 0.80 | 0.60-1.20 |
| Verhoeff – Media – Elastic Fibers % | 13 | 41.69 | 21.79 | 8.00 | 69.00 | 38.00 | 22.00-64.00 |
| Masson – All layers – Fibrosis % | 15 | 40.53 | 14.11 | 22.00 | 74.00 | 39.00 | 29.50-47.50 |
| **IMUNOHISTOCHEMICAL VARIABLES** |  |  |  |  |  |  |  |
| Actina - Tunica media % | 10 | 21.22 | 12.10 | 4.40 | 41.00 | 17.00 | 13.25-31.25 |
| CD20 - Intima layer - PC | 15 | 78.40 | 140.81 | 5.00 | 513.00 | 20.00 | 8.00-50.00 |
| CD20 - Tunica media - PC | 15 | 106.20 | 336.84 | 1.00 | 1320.00 | 5.00 | 3.50-32.00 |
| CD20 - Adventitia layer - PC | 15 | 720.53 | 1050.48 | 41.00 | 3497.00 | 320.00 | 79.50-754.50 |
| CD45 - Intima layer - PC | 15 | 581.73 | 2101.04 | 1.00 | 8175.00 | 23.00 | 7.00-68.00 |
| CD45 - Tunica media - PC | 15 | 93.800 | 199.57 | 1.00 | 773.00 | 24.00 | 8.00-57.50 |
| CD45 - Adventitia layer - PC | 15 | 1223.13 | 1417.82 | 25.00 | 4456.00 | 409.00 | 252.00-2010.50 |
| CD68 - Intima layer - PC | 14 | 381.57 | 691.72 | 3.00 | 2423.00 | 38.00 | 18.50-405.25 |
| CD68 - Tunica media - PC | 14 | 113.43 | 237.27 | 1.00 | 846.00 | 21.00 | 2.75-67.25 |
| CD68 - Adventitia layer - PC | 14 | 665.00 | 747.81 | 12.00 | 2480.00 | 374.00 | 118.00-1035.75 |
| PPARgama - Intima layer - PC | 14 | 1391.50 | 2119.56 | 6.00 | 5279.00 | 230.00 | 47.25-1800.00 |
| PPARgama - Tunica media - PC | 14 | 291.07 | 389.65 | 9.00 | 1248.00 | 85.50 | 23.25-481.50 |
| PPARgama - Adventitia layer - PC | 14 | 2903.14 | 4778.69 | 59.00 | 17541.00 | 778.00 | 167.25-3787.25 |
| KLF5 - Intima layer - PC | 14 | 501.07 | 565.72 | 32.00 | 1715.00 | 283.50 | 124.25-508.50 |
| KLF5 - Tunica media - PC | 14 | 39.57 | 50.13 | 2.00 | 197.00 | 26.00 | 9.25-47.75 |
| KLF5 - Adventitia layer - PC | 14 | 2291.57 | 3066.50 | 504.00 | 12233.00 | 1013.50 | 606.25-2514.50 |
| MMP2 - Intima layer - PC | 14 | 643.07 | 639.51 | 15.00 | 2146.00 | 385.00 | 174.50-1053.00 |
| MMP2- Tunica media - PC | 14 | 173.43 | 212.18 | 1.00 | 656.00 | 99.00 | 34.25-181.00 |
| MMP2- Adventitia layer - PC | 14 | 2979.57 | 2062.43 | 1090.00 | 7311.00 | 2517.00 | 1316.25-3614.00 |

n: sample size; %: percentage; mm: millimeters; SD: standard deviation; PC: positive cells count per mm^2^; Min: minimum value; Max: maximum value; IQR: interquartile range (Q1-Q3); SAH: Systemic Arterial Hypertension; DM: Diabetes Mellitus.
